# Comparison of reconstruction plate screw fixation and percutaneous cannulated screw fixation in treatment of Tile B1 type pubic symphysis diastasis: a finite element analysis and 10-year clinical experience

**DOI:** 10.1186/s13018-015-0272-y

**Published:** 2015-09-22

**Authors:** Ke-He Yu, Jian-Jun Hong, Xiao-Shan Guo, Dong-Sheng Zhou

**Affiliations:** Department of Traumatic Orthopedics, Shandong Provincial Hospital, Shandong University, No. 324 Jin Wu Wei Seventh Road, Jinan, 250021 Shandong China; Department of Orthopedics, The Second Affiliated Hospital of Wenzhou Medical University, 109# XueYuan Western Road, Wenzhou, Zhejiang 325000 China

**Keywords:** Pubic symphysis diastasis, Cannulated screw, Reconstruction plate, Finite element analysis, Comparative study

## Abstract

**Objective:**

The objective of this study is to compare the biomechanical properties and clinical outcomes of Tile B1 type pubic symphysis diastasis (PSD) treated by percutaneous cannulated screw fixation (PCSF) and reconstruction plate screw fixation (RPSF).

**Materials and Methods:**

Finite element analysis (FEA) was used to compare the biomechanical properties between PCSF and RPSF. CT scan data of one PSD patient were used for three-dimensional reconstructions. After a validated pelvic finite element model was established, both PCSF and RPSF were simulated, and a vertical downward load of 600 N was loaded. The distance of pubic symphysis and stress were tested. Then, 51 Tile type B1 PSD patients (24 in the PCSF group; 27 in the RPSF group) were reviewed. Intra-operative blood loss, operative time, and the length of the skin scar were recorded. The distance of pubic symphysis was measured, and complications of infection, implant failure, and revision surgery were recorded. The Majeed scoring system was also evaluated.

**Results:**

The maximum displacement of the pubic symphysis was 0.408 and 0.643 mm in the RPSF and PCSF models, respectively. The maximum stress of the plate in RPSF was 1846 MPa and that of the cannulated screw in PCSF was 30.92 MPa. All 51 patients received follow-up at least 18 months post-surgery (range 18–54 months). Intra-operative blood loss, operative time, and the length of the skin scar in the PCSF group were significantly different than those in the RPSF group. No significant differences were found in wound infection, implant failure, rate of revision surgery, distance of pubic symphysis, and Majeed score.

**Conclusion:**

PCSF can provide comparable biomechanical properties to RPSF in the treatment of Tile B1 type PSD. Meanwhile, PCSF and RPSF have similar clinical and radiographic outcomes. Furthermore, PCSF also has the advantages of being minimally invasive, has less blood loss, and has shorter operative time and skin scar.

## Introduction

With the increased occurrence of high-energy injuries caused by traffic accidents or falling from high places, the incidence rate of pelvic and acetabular fractures and economic burden is increasing [1–3]. It has been reported that the pubic symphysis diastasis (PSD) is approximately 24 % in pelvic fractures [4].

For PSD, open reduction and reconstruction plate screw fixation (RPSF) is the primary technique currently used [5, 6]. However, traditional open surgery for pelvic fractures has many disadvantages, such as considerable trauma of surrounding tissues and intra-operative blood loss [7, 8].

With the development of the intra-operative imaging system and the improvement of surgical instruments, many different types of minimally invasive techniques have been reported to treat pelvic fractures [9–11] and have advantages such as shorter skin scar, less blood loss, and less soft tissue trauma. Available reports [12, 13] about percutaneous cannulated screw fixation (PCSF) for PSD are still rare, and most of these patients have the combined trauma of PSD with other site trauma/fractures of the pelvis, which influences the evaluation of the outcomes of PCSF in the treatment of PSD. The biomechanical properties and clinical outcomes of PSD treated by PCSF remain unclear and are controversial. According to the Tile classification of pelvic disruption [14], type B1 is an “open book” lesion. Further, type B1 is considered to be simple PSD with rotational instability, and it is one of the indications for both PCSF and RPSF [12] and is useful for comparing the difference in the biomechanical properties and clinical outcomes of the above-mentioned surgical techniques.

## Materials and methods

In this study, finite element analysis was performed to compare the biomechanical properties of PCSF to RPSF in the treatment of PSD. After then, we retrospectively reviewed the prospectively collected data for PCSF and RPSF in the treatment of Tile B1 PSD between January 2003 and December 2012.

This study was performed following the Declaration of Helsinki principles and was approved by the Institutional Review Board (IRB) of The Second Affiliated Hospital of Wenzhou Medical University. Informed consent was obtained from all participants.

## Part of finite element analysis

**Fig. 1:**
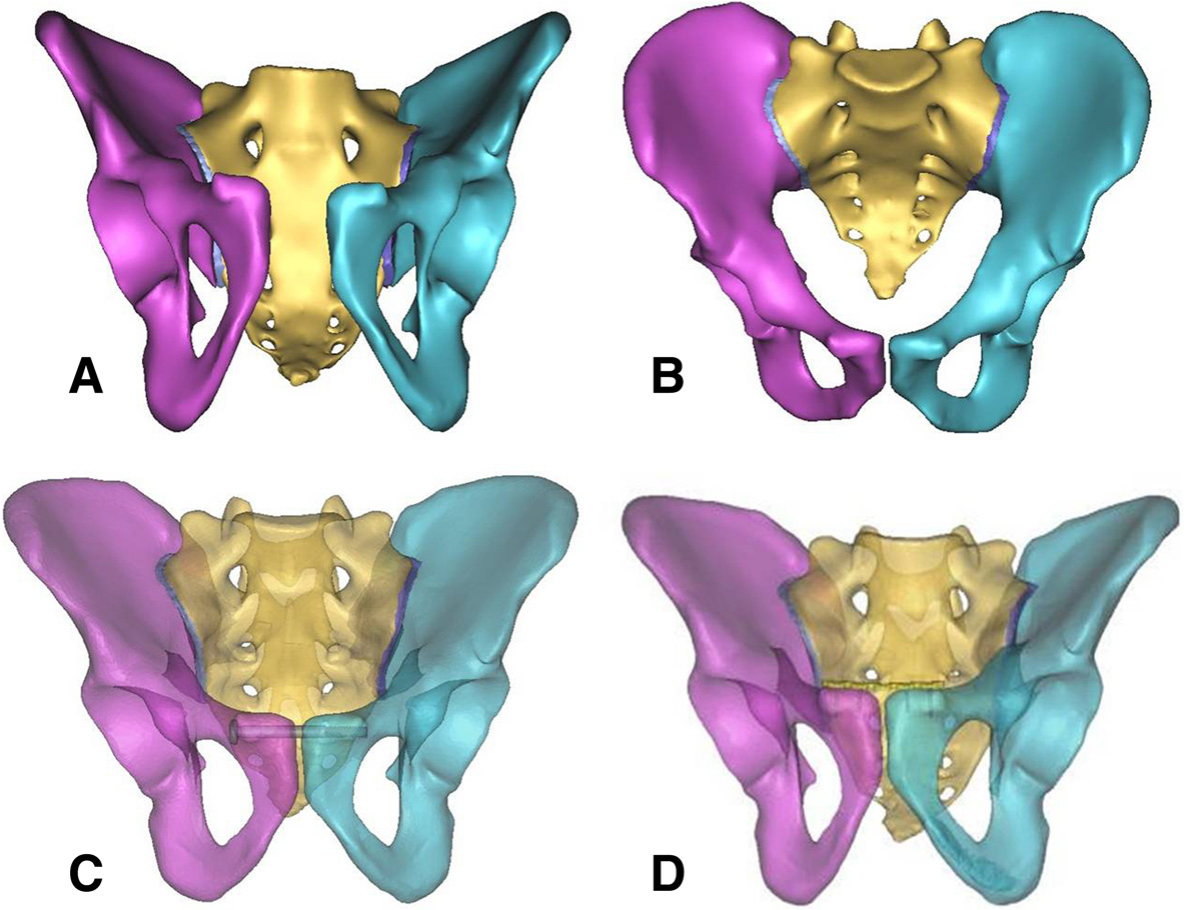
The PSD model was reconstructed on the basis of the raw data using Mimics 14.11 software and received reduction through the virtual operation function in the Mimics software. **a** Three-dimensional reconstruction of PSD using software Mimics. **b** The virtual surgery simulation function of software Mimics was used to simulate reduction of PSD. **c** Simulating the cannulated screw fixation in the treatment of PSD. **d** Simulating the reconstruction plate internal fixation for PSD

The CT scan data in the DICOM format of one of the PSD patients was imported into Mimics V14.11 software for three-dimensional reconstructions and to simulate the reduction of PSD (Fig. 1a, b). Cannulated screw and plate-screw models were established on CATIA. All of the parts were imported into ABAQUS 6.11 for assembling and meshing (nodes and element number of each model are shown in Table 1). The property of bone material was assigned in Mimics according to the gray value of the CT image, and 100 materials were assigned. The material formula was as follows: *ρ* = 1.122*HU + 47(g/cm^3^), *E* = 1.92*ρ − 170 (MPa). Material properties of organizations according to previous literatures [15–21] are shown in Table 2, and the properties of ligaments are shown in Table 3.

**Table 1:** Number of nodes and elements in series of FE models with pelvises

Part	Nodes	Element number
Left ilium	63,500	322,214
Right ilium	40,508	205,408
Left sacroiliac joint	78,380	399,129
Right sacroiliac joint	79,319	404,146
Sacrum	72,696	375,426
Cannulated screw	16,145	75,674
Plate + screw	65,354	324,089

**Table 2:** Material property of series of FE models [12–18]

Materials	Elastic modulus	Poisson ratio	Friction coefficient
	*E* (MPa)	*μ*	*f*
Titanium plate	110,000	0.30	0.45
Titanium screw	110,000	0.30	–
Articular cartilage	11.9–0.48	0.40	0.0024 ~ 0.24
Cortical bone	17,000	0.3	0.4
Cancellous bone	129	0.2	0.4

**Table 3:** Property of ligaments of FE model

Ligaments	K (N/mm)	Number of springs
Anterior sacroiliac	1500	30
Long posterior sacroiliac	5000	16
Short posterior sacroiliac	8000	25
Interspinous	4000	21
Superior pubic	250	12
Arcuate pubic	250	12
Sacrospinous	5000	16
Sacrotuberous	9000	16

### Simulation of two operational fixation models for PSD

RPSF and PCSF were simulated to fix the PSD. A cannulated screw is a short-thread, hollow nail with a diameter of 6.5 mm, while a reconstruction plate-screw system has five holes on the reconstruction titanium plate and a screw with a diameter of 3.5 mm. The reconstruction plate and cannulated screw were merged with the ilium, sacrum, and sacroiliac joint cartilage, via the Boolean operation, which generated the fixed model for the separation of the symphysis pubis. Simulation was performed as follows: (1) A cannulated screw fixed the contralateral pubic through one-side pubic nodules (Fig. 1c). (2) A reconstruction plate was put on the anterior and superior border of the symphysis pubis, and two screws were put on two sides of the titanium plate for fixation (Fig. 1d).

### Contact, constraint, and load of three-dimensional finite element model

In the research, the contact relation between the ilium, sacrum, and sacroiliac joint was set as binding constraints, as same as the contact relation between screw and bones. The contact relation between plate and bone was set as sliding friction. In reference to previous studies [22–24], a vertical downward load of 600 N was imposed on the surface of the sacrum to simulate the gravity of the upper part of the body.

## Part of clinical comparative study

Fifty-one Tile type B1 PSD patients (open book lesion), including 24 who were treated by PCSF (PCSF group) and 27 who were treated by open reduction and RPSF (RPSF group), were reviewed in this study. The patients’ basic information, i.e., age and gender, intra-operative blood loss, operative time, and length of the skin scar were recorded. The distance of pubis was measured at pre-operation, 3 months post-operation, and final follow-up at the PACS System (INFINITT, Seoul, South Korea), which was widely used to measure the distance and area on radiographic images and is very convenient and accurate [25, 26]; in this study, only the minimized horizontal distance of pubis was measured (Fig. 4). Complications of infection, implant failure, and revision surgery were recorded to evaluate the safety of the above-mentioned surgical techniques. The Majeed scoring system [27] was used to assess functional outcomes.

### Surgical technique

In the PCSF group, patients were placed in the supine position, and high-resolution anteroposterior, outlet and inlet views were obtained by C-arm X-ray fluoroscopy monitoring. Two Schanz pins were inserted into bilateral iliac crests to assist with the reduction, and then, a large towel-clip clamp or Weber clamp was used across the pubic symphysis to manually reduce the PSD. After satisfactory reduction was achieved, K-wire was introduced at the point between the pubic tubercle and superior ramus of the pubis at one side and was forwarded to the other side of the pubic symphysis. Caution should be taken to avoid injuring the spermatic cord in males and the round ligament of the uterus in females. A cannulated compression screw was then introduced along the K-wire; to decrease the risk of screw pull out and to produce compression, the screw thread must go beyond the contralateral cortex. In the RPSF group, all patients were placed in the supine position, and a midline vertical incision was made. The plate and screws were introduced via the traditional open technique [5, 12].

## Statistical analysis

The data were analyzed with SPSS software (version 17.0, SPSS Inc., Chicago, IL). Data regarding the distance of diastasis at pre-operation, 3 months post-operation, and final follow-up were tested by a one-way repeated-measures analysis of variance (ANOVA), and the differences in blood loss, operative time, and length of skin scar between these two different surgical techniques were tested by a two-sample *t* test. Complications of infection and implant failure, as well as the rate of revision surgery, were tested using a Chi-squared test. The Majeed score was compared using the Mann-Whitney *U* test. A *P* value of <0.05 was considered significant.

## Results

### Finite element analysis

#### Analysis of the whole stress

**Fig. 2:**
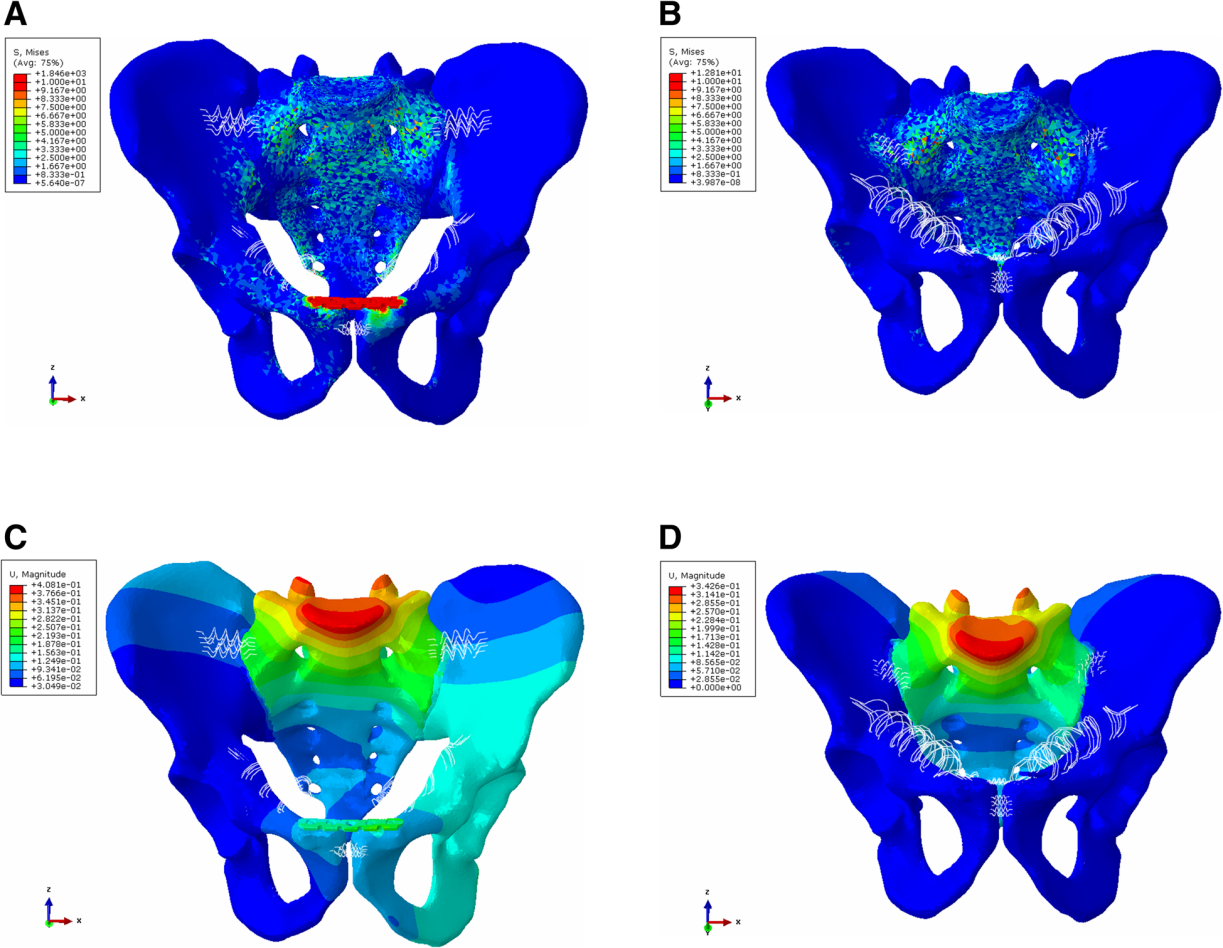
The stress distribution around the pubic symphysis after RPSF (**a**) and PCSF (**b**). The displacement nephogram after fixation with RPSF (**c**) showed that there was no obvious displacement in the position of the pubic symphysis, and similarly, there was no evident displacement with PCSF (**d**)

The maximum whole stress was 180.8 MPa when RPSF was used for the treatment of PSD (Fig. 2a), whereas the maximum whole stress was 12.8 MPa when PCSF was used (Fig. 2b). The stress of the two surgical methods was mainly distributed on the sacrum after fixation. The average von Mises stress of the pelvis is shown in Table 4.

**Table 4:** The von Mises stress and displacement of pelvis

	Anterior ring			Posterior ring		
	RPSF	PCSF	(RPSF − PCSF)/RPSF	RPSF	PCSF	(RPSF − PCSF)/RPSF
The von Mises stress (MPa)	[0.212–1.132]	[0.336–1.004]	−58.49~11.31 %	[0.801–3.122]	[0.787–1.989]	17.48~36.29 %
The displacement (mm)	[0.051–0.201]	[0.047–0.059]	7.84~70.65 %	[0.031–0.153]	[0.056–0.097]	−80.6~36.6 %

*PCSF* percutaneous cannulated screw fixation, *RPSF* reconstruction plate screw fixation

#### Displacement of the pelvis

The whole maximum displacement of the bilateral pelvis (two maximum displacement points of the bilateral pelvis at each finite element analysis (FEA) model) at the RPSF FEA model was 0.408 mm (Fig. 2c), whereas that at the PCSF FEA model was 0.643 mm (Fig. 2d), which indicated that both treatment methods can effectively repair separation of the symphysis pubis. The displacement of the pelvis is shown in Table 4.

#### Stress analysis of cannulated screw and plate

**Fig. 3:**
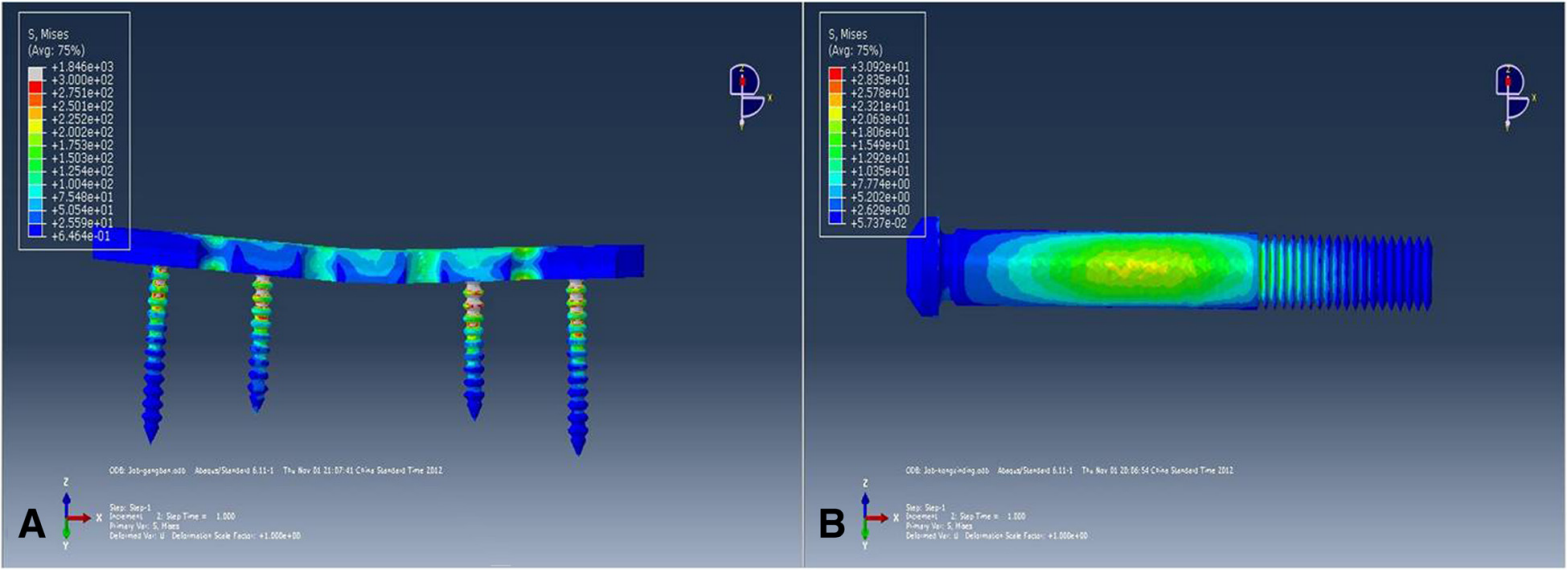
Stress nephogram of the plate and cannulated screw. **a** The maximum stress of the plate was 1846 MPa. **b** The maximum stress of the cannulated screw was 30.92 MPa

The maximum stress of the plate was 1846 MPa (Fig. 3a), while the maximum stress of the cannulated screw was 30.92 MPa (Fig. 3b), which was much less than the plate.

## Clinical and radiographic outcomes

**Fig. 4:**
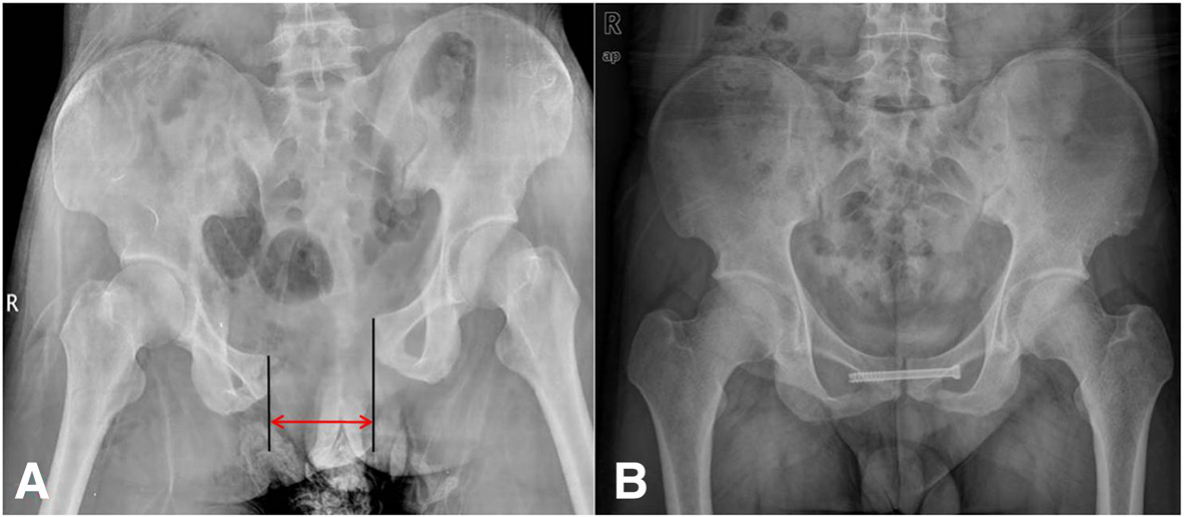
The radiographic images showed that one Tile B1 PSD patient (**a**) was treated with closed reduction and percutaneous cannulated screw fixation (**b**)

All 51 patients received follow-up at least 18 months, range 18–54 months (29.4 ± 8.8 months) (Fig. 4), post-surgery. The results showed that intra-operative blood loss, operative time, and the length of the skin scar in the PCSF group were significantly less than those in the RPSF group. Data are shown in Table 5.

**Table 5:** The results of blood loss, operative time, and skin scar between two groups (Mean ± SD)

	PCSF group	RPSF group	*T*	*P* value
Number	24	27	–	–
Age	33.4 ± 9.1	34.8 ± 11.7	–	–
Gender	15 males, 9 females	19 males, 8 females	–	–
Operative time (min)	26.3 ± 5.9	68.9 ± 13.6	−14.771	0.000
Intra-operative blood loss (ml)	9.6 ± 5.7	171.9 ± 68.3	−12.294	0.000
Length of skin scar (cm)	1.8 ± 0.6	8.1 ± 1.1	−24.864	0.000

*PCSF* percutaneous cannulated screw fixation, *RPSF* reconstruction plate screw fixation

The distance of pubic symphysis was 47.6 ± 14.2 mm in the PCSF group and 43.5 ± 11.3 mm in the RPSF group at pre-operation, and these values decreased to 4.6 ± 1.1 and 4.5 ± 1.0 mm, respectively, 3 months post-operation (*P* = 0.000). The distances were maintained at 4.8 ± 1.2 and 4.5 ± 1.2 mm, respectively, at the final follow-up (Table 6).

**Table 6:** The distances of pubic symphysis between the PCSF and RPSF groups (mm)

	Pre-operation	3 months after operation	Final follow-up
PCSF group	47.58 ± 14.24	4.63 ± 1.06^*^	4.84 ± 1.21^**^
RPSF group	43.52 ± 11.31	4.55 ± 1.04^***^	4.53 ± 1.16^****^
*T* value	1.135	0.261	0.928
*P* value	0.262	0.795	0.358

Compared to pre-operation, both the distance of pubic symphysis in the PCSF and RPSF groups were significantly decreased at 3 months after operation (*P = 0.000, ***P = 0.000), and the reduction of post-operation was maintained at the final follow-up (**P = 0.928, ****P = 0.942). Comparisons between the PCSF and RPSF groups in all pre-operation, 3 months after operation, and final follow-up time points did not have significant difference

One case of wound infection was found regarding the RPSF group. In both the PCSF and RPSF groups, two cases of implant failure were observed, as was one case of revision surgery in each group. No significant difference was found regarding complications, implant failure, and revision surgery between the PCSF and RPSF groups (Table 7). The Majeed scores of both groups at the final follow-up are shown in Table 8, and no statistically significant difference was found between the groups.

**Table 7:** The results of wound infection, implant failure, and revision surgery

	PCSF group	RPSF group	*P*
	(*N* = 24)	(*N* = 27)	
Wound infection^a^	1	2	1.000
Implant failure^a^	2	2	1.000
Revision surgery^b^	1	1	1.000

*PCSF* percutaneous cannulated screw fixation, *RPSF* reconstruction plate screw fixation

^a^Pearson’s corrected *χ* ^2^ test

^b^Fisher’s exact test

**Table 8:** Functional results of the Majeed scoring system (Mann-Whitney *U* test)

	Excellent	Good	Fair	Poor	*P*
	(>85)	(70–84)	(55–69)	(<55)	
PCSF group	18	5	1	0	0.814
RPSF group	18	7	2	0	

*PCSF* percutaneous cannulated screw fixation, *RPSF* reconstruction plate screw fixation

## Discussion

The pelvic fracture, which mainly results from high-energy injuries, is well-known for having a high disability rate and associated mortality. The mortality rate following pelvic fractures ranges from 5 to 20 % [28–30] which is a remaining challenge in the field of orthopedics. PSD can be observed with other site fractures of the pelvis, or it can occur alone. Simple PSD is the type B1 lesion, according to the Tile classification of pelvic disruption [14], and is named the “open book” lesion, which is rotationally unstable. If the distance of symphysis pubis was more than 25 mm in plain radiography, the anterior sacroiliac ligaments are mostly damaged, and surgical intervention was recommended [31].

Traditional open reduction and RPSF have been widely used for PSD [8, 22, 32]. Mu et al. [13], Chen et al. [12], and Taller et al. [33] reported using PCSF in the treatment of PSD. However, in previous literatures, most of the PSD patients had combined trauma of sites in the pelvis, which influences the evaluation of outcomes. In this study, only the Tile B1 type patients were included. However, Tile B1 type is not usual, and over the past 10 years, 51 such patients have been treated in our department. The biomechanical finite element properties of PCSF and RPSF in the treatment of type B1 PSD were also compared.

As part of the FEA, all mechanical parameters, density, Poisson ratio, and elastic modulus were used according to previous literatures to establish a precise pelvic FEA model [15, 19, 34, 35]. Mimics software was used to convert different gray values into corresponding densities and to calculate the Poisson ratio and elastic modulus, which makes the pelvic model closer to the substance and the analytical results more accurate.

Cano-Luis et al. [24] compared the biomechanical properties of the cannulated screw and fixation with PSD and intact specimens. Ten specimens of fresh human cadavers were used. The researchers found that there was no significant difference in the average displacement (mm) between the intact pubic symphysis and PSD fixed by cannulated screw (*P* > 0.7) after application of an axial load of 300 N, but a significant difference was observed between the average displacements of the PSD model and PSD fixed by cannulated screw (*P* < 0.05). Their biomechanical studies *in vitro* supported the idea that cannulated screws have the ability to resist rotational forces. In this FEA, we found that the maximum displacement of the plate was 0.408 mm and of the cannulated screw was 0.643 mm at a vertical downward force of 600 N. Both the PCSF and RPSF groups showed that they can provide rigid support fixation. The stress analysis showed that the maximum stress of the plate was 1846 MPa, which was significantly higher than that of the cannulated screw (30.92 MPa). This biomechanical benefit of the cannulated screw can be attributed to the intramedullary fixation of a cannulated screw. It had already proven that intramedullary nailing could decrease the amount of stress burden of the implant and had lower failure rate in long bone fractures than the plate [36]. The screw-plate contact site in the screw-plate and on the middle of the cannulated screw, where the stress was concentrated, were the exact sites where caution had to be taken to avoid implant failure.

The clinical data of 24 patients treated by PCSF (PCSF group) and 27 patients treated by open reduction and RPSF (RPSF group) were compared. We found that both the PCSF and RPSF techniques can significantly reduce the distance of PSD and have a similar result of functional outcome. No significant difference was calculated between them. Wound infection was observed in one case in the PCSF group and in two cases in the RPSF group, but without significant difference, and no significant difference was observed in implant failure and revision surgery. However, we found that the PCSF technique has advantages, including less intra-operative blood loss and shorter operative time and skin scar. Our results were similar to Mu et al. [13] and Chen et al. [12].

Although there are some minimally invasive advantages of the PCSF technique, it still has many limitations and is not widely used. We suppose that three reasons may influence the use of this technique, Firstly, PCSF, as a novel minimally invasive technique, is challenging to the surgeons, and there is a learning curve [37]. Secondly, the procedure of the PCSF technique involves intra-operative C-arm X-ray fluoroscopy monitoring, and radiation exposure may increase the risk of cancer [38, 39]; therefore, some surgeons are unwilling to perform the percutaneous technique. Thirdly, the medical insurance policy may also influence what technique a surgeon chooses to use. In China, the PCSF surgery is covered by the government medical insurance, and the patients who underwent this surgery can reimburse their medical cost, therefore encourage some Chinese surgeons to perform this surgery. The PCSF technique also has contraindications. Mu et al. [13] suggested that patients with surgical site skin infection, bladder injury, or open trauma wound are not suitable for the PCSF technique.

## Conclusion

PCSF can provide comparable biomechanical properties to RPSF in the treatment of Tile B1 type PSD. Meanwhile, PCSF and RPSF have similar clinical and radiographic outcomes. Furthermore, PCSF also has the advantages of being minimally invasive, has less blood loss, and has shorter operative time and skin scar.

## References

[1] Ragnarsson B, Jacobsson B (1992). Epidemiology of pelvic fractures in a Swedish county. Acta Orthop Scand.

[2] Burge R, Dawson-Hughes B, Solomon DH, Wong JB, King A, Tosteson A (2007). Incidence and economic burden of osteoporosis-related fractures in the United States, 2005–2025. J Bone Miner Res.

[3] Inaba K, Sharkey PW, Stephen DJ, Redelmeier DA, Brenneman FD (2004). The increasing incidence of severe pelvic injury in motor vehicle collisions. Injury.

[4] Pohlemann T, Bosch U, Gansslen A, Tscherne H (1994). The Hannover experience in management of pelvic fractures. Clin Orthop Relat Res.

[5] Putnis SE, Pearce R, Wali UJ, Bircher MD, Rickman MS (2011). Open reduction and internal fixation of a traumatic diastasis of the pubic symphysis: one-year radiological and functional outcomes. J Bone Joint Surg Br.

[6] Lange RH, Hansen ST (1985). Pelvic ring disruptions with symphysis pubis diastasis. Indications, technique, and limitations of anterior internal fixation. Clin Orthop Relat Res.

[7] Giannoudis PV, Chalidis BE, Roberts CS (2008). Internal fixation of traumatic diastasis of pubic symphysis: is plate removal essential?. Arch Orthop Trauma Surg.

[8] Sagi HC, Papp S (2008). Comparative radiographic and clinical outcome of two-hole and multi-hole symphyseal plating. J Orthop Trauma.

[9] Farouk O, Kamal A, Badran M, El-Adly W, El-Gafary K (2014). Minimal invasive para-rectus approach for limited open reduction and percutaneous fixation of displaced acetabular fractures. Injury.

[10] Sharma A, Jain PK, Shaw CJ, Sedman PC (2004). Successful laparoscopic repair of a traumatic pubic symphysis hernia. Surg Endosc.

[11] Routt ML, Nork SE, Mills WJ (2000). Percutaneous fixation of pelvic ring disruptions. Clin Orthop Relat Res.

[12] Chen L, Zhang G, Song D, Guo X, Yuan W (2012). A comparison of percutaneous reduction and screw fixation versus open reduction and plate fixation of traumatic symphysis pubis diastasis. Arch Orthop Trauma Surg.

[13] Mu WD, Wang H, Zhou DS, Yu LZ, Jia TH, Li LX (2009). Computer navigated percutaneous screw fixation for traumatic pubic symphysis diastasis of unstable pelvic ring injuries. Chin Med J (Engl).

[14] Tile M (1988). Pelvic ring fractures: should they be fixed?. J Bone Joint Surg Br.

[15] Zhang L, Yang G, Wu L, Yu B (2010). The biomechanical effects of osteoporosis vertebral augmentation with cancellous bone granules or bone cement on treated and adjacent non-treated vertebral bodies: a finite element evaluation. Clin Biomech (Bristol, Avon).

[16] Vleeming A, Volkers AC, Snijders CJ, Stoeckart R (1990). Relation between form and function in the sacroiliac joint. Part II: biomechanical aspects. Spine (Phila Pa 1976).

[17] Dalstra M, Huiskes R, Odgaard A, van Erning L (1993). Mechanical and textural properties of pelvic trabecular bone. J Biomech.

[18] Zuo Z (2006). Three-dimensional finite element analysis and biomechanics of sacroiliac complex, Medical Doctorship Thesis.

[19] Dalstra M, Huiskes R, van Erning L (1995). Development and validation of a three-dimensional finite element model of the pelvic bone. J Biomech Eng.

[20] Mechlenburg I, Nyengaard JR, Gelineck J, Soballe K (2007). Cartilage thickness in the hip joint measured by MRI and stereology—a methodological study. Osteoarthritis Cartilage.

[21] Zhang L, Peng Y, Du C, Tang P (2014). Biomechanical study of four kinds of percutaneous screw fixation in two types of unilateral sacroiliac joint dislocation: a finite element analysis. Injury.

[22] Simonian PT, Routt ML, Harrington RM, Tencer AF (1994). Box plate fixation of the symphysis pubis: biomechanical evaluation of a new technique. J Orthop Trauma.

[23] Varga E, Hearn T, Powell J, Tile M (1995). Effects of method of internal fixation of symphyseal disruptions on stability of the pelvic ring. Injury.

[24] Cano-Luis P, Giraldez-Sanchez MA, Martinez-Reina J, Serrano-Escalante FJ, Galleguillos-Rioboo C, Lazaro-Gonzalvez A (2012). Biomechanical analysis of a new minimally invasive system for osteosynthesis of pubis symphysis disruption. Injury.

[25] Wu AM, Wang XY, Zhao HZ, Lin SL, Xu HZ, Chi YL (2014). An imaging study of the compressed area, bony fragment area, and the total fracture-involved area in thoracolumbar burst fractures. J Spinal Disord Tech.

[26] Tian NF, Xu HZ, Wang XY, Chen QJ, Zheng LC (2010). Morphometric comparisons between the pedicle and the pedicle rib unit in the immature Chinese thoracic spine: a computed tomographic assessment. Spine (Phila Pa 1976).

[27] Majeed SA (1989). Grading the outcome of pelvic fractures. J Bone Joint Surg Br.

[28] Fitzgerald CA, Morse BC, Dente CJ (2014). Pelvic ring fractures: has mortality improved following the implementation of damage control resuscitation?. Am J Surg.

[29] Hauschild O, Strohm PC, Culemann U, Pohlemann T, Suedkamp NP, Koestler W (2008). Mortality in patients with pelvic fractures: results from the German pelvic injury register. J Trauma.

[30] Pohlemann T, Tscherne H, Baumgartel F, Egbers HJ, Euler E, Maurer F (1996). Pelvic fractures: epidemiology, therapy and long-term outcome. Overview of the multicenter study of the Pelvis Study Group. Unfallchirurg.

[31] Young JW, Burgess AR, Brumback RJ, Poka A (1986). Pelvic fractures: value of plain radiography in early assessment and management. Radiology.

[32] Webb LX, Bosse MJ, Mayo KA, Lange RH, Miller ME, Swiontkowski MF (1990). Results in patients with craniocerebral trauma and an operatively managed acetabular fracture. J Orthop Trauma.

[33] Taller S, Lukas R, Sram J (2011). Single cannulated screws for stabilisation of pelvic ring and acetabular fractures. Acta Chir Orthop Traumatol Cech.

[34] Anderson AE, Peters CL, Tuttle BD, Weiss JA (2005). Subject-specific finite element model of the pelvis: development, validation and sensitivity studies. J Biomech Eng.

[35] Phillips AT, Pankaj P, Howie CR, Usmani AS, Simpson AH (2007). Finite element modelling of the pelvis: inclusion of muscular and ligamentous boundary conditions. Med Eng Phys.

[36] Virkus WV, Goldberg SH, Lorenz EP (2008). A comparison of compressive force generation by plating and intramedullary nailing techniques in a transverse diaphyseal humerus fracture model. J Trauma.

[37] Rommens PM (2007). Is there a role for percutaneous pelvic and acetabular reconstruction?. Injury.

[38] Mastrangelo G, Fedeli U, Fadda E, Giovanazzi A, Scoizzato L, Saia B (2005). Increased cancer risk among surgeons in an orthopaedic hospital. Occup Med (Lond).

[39] Singer G (2005). Occupational radiation exposure to the surgeon. J Am Acad Orthop Surg.

